# Quantitative Assessment of Treatment Response in Metastatic Breast Cancer Patients by SPECT-CT Bone Imaging—Getting Closer to PET-CT

**DOI:** 10.3390/cancers15030696

**Published:** 2023-01-23

**Authors:** Mirela Gherghe, Mario-Demian Mutuleanu, Adina Elena Stanciu, Ionela Irimescu, Alexandra Maria Lazar, Radu Valeriu Toma, Oana Gabriela Trifanescu, Rodica Maricela Anghel

**Affiliations:** 1 Nuclear Medicine Department, University of Medicine and Pharmacy Carol Davila Bucharest, 050474 București, Romania; 2 Nuclear Medicine Department, Institute of Oncology “Prof. Dr. Alexandru Trestioreanu”, 022328 Bucharest, Romania; 3 Carcinogenesis and Molecular Biology Department, Institute of Oncology “Prof. Dr. Alexandru Trestioreanu”, 022328 Bucharest, Romania; 4 Oncology Department, University of Medicine and Pharmacy Carol Davila Bucharest, 050474 Bucharest, Romania; 5 Radiotherapy I Department, Institute of Oncology “Prof. Dr. Alexandru Trestioreanu”, 022328 Bucharest, Romania; 6 Radiotherapy II Department, Institute of Oncology “Prof. Dr. Alexandru Trestioreanu”, 022328 Bucharest, Romania

**Keywords:** quantitative analysis, SPECT-CT, metastatic bone disease, qualitative imaging interpretation

## Abstract

**Simple Summary:**

The aim of our study is to demonstrate the added value of the quantitative interpretation of SPECT-CT images compared to qualitative assessment regarding changes in radiotracer uptake between the baseline and follow-up scans in breast cancer patients treated for metastatic bone disease. By implementing this method of imaging interpretation, a more standardized and robust approach of patient follow-up evaluation can be obtained, which is particularly important for research purposes.

**Abstract:**

Background: Cancer represents the major cause of death mainly through its ability to spread to other organs, highlighting the importance of metastatic disease diagnosis and accurate follow up for treatment management purposes. Although until recently the main method for imaging interpretation was represented by qualitative methods, quantitative analysis of SPECT-CT data represents a viable, objective option. Methods: Seventy-five breast cancer patients presenting metastatic bone disease underwent at least two Bone SPECT-CT studies using [^99m^Tc]-HDP between November 2019 to October 2022. Results: Our findings show a good positive relationship between the qualitative methods of imaging interpretation and quantitative analysis, with a correlation coefficient of 0.608 between qualitative whole body scintigraphy and quantitative SPECT-CT, and a correlation coefficient of 0.711 between the qualitative and quantitative interpretation of SPECT-CT data; nevertheless, there is a need for accurate, objective and reproducible methods for imaging interpretation, especially for research purposes. Conclusions: Quantitative evaluation of the SPECT-CT data has the potential to be the first choice of imaging interpretation for patient follow up and treatment response evaluation, especially for research purposes, because of its objectivity and expression of uptake changes in absolute units.

## 1. Introduction

Cancer represents one of the major causes of death worldwide, mainly through its ability to spread to other organs [1]. Certain types of cancer, such as breast and prostate, are more likely to spread to the bone [2]. Therefore, metastatic bone disease is seen in approximately 65–75% of stage IV cancer patients [3]. It is well known that bone metastases typically occur through hematogenous dissemination [4]. This explains why the most common sites for bone metastases are represented by bones rich in red marrow, such as the vertebrae, the ribs, the pelvis, and the epiphysis of long bones. In contrast, metastases in the appendicular skeleton are rarely seen [5]. To better understand the bone metastasis mechanism, it is important to consider the particularities of the bone microenvironment regarding bone remodeling performed by osteoblasts and osteoclasts. Tumor cells produce various biological factors that stimulate not only osteoclast bone resorption and synthesis, which influences bone volume, but also the growth of metastatic cancer cells in bone [6]. Thus, early identification and follow-up of patients with bone metastases are of great importance for appropriate treatment selection and therapy response evaluation [2,6,7]. Molecular imaging techniques such as whole body scintigraphy (WBS) and, in particular, hybrid imaging, represented by single photon emission computed tomography-computed tomography (SPECT-CT) or positron emission tomography-computed tomography (PET-CT) scanners, have an increasing role in bone metastases detection and treatment response evaluation [8,9,10]. Although WBS has been used for many years to detect bone metastases [11], hybrid imaging techniques have led to improved diagnostic accuracy [12]. Although PET-CT using 18fluorine-fluorodeoxyglucose ([^18^F]-FDG), [^18^F]-sodium-fluoride (NaF) or [^18^F]-fluorometylcholine (FCH) are available for detecting bone metastases with higher accuracy a than conventional bone scan [13,14,15,16], the process of using PET-CT for metastatic bone lesion assessment is restricted by the lack of funding, availability of PET-CT scanners and national reimbursement strategy [17,18]. Both SPECT-CT and PET-CT imaging modalities require the administration of radiopharmaceuticals and are associated with anatomic imaging techniques such as CT [19]. In clinical practice, qualitative evaluation represents the main method used for imaging interpretation. However, hybrid imaging has led to the emergence of quantitative interpretation methods by expressing the radiotracer activity seen on the acquired images in absolute units, thus providing a more standardized evaluation method [20,21,22]. Until recently, quantification methods were possible only on PET-CT studies, but recent technological development and new reconstruction algorithm techniques have made quantification easier and more accurate, which has enabled its further expansion to SPECT-CT, resulting in radiotracer activity interpretation in terms of standardized uptake value (SUV) [21,23,24,25]. The reduced costs, wide availability and continued growth in SPECT-CT imaging techniques indicate that SPECT-CT can be a viable option in performing quantitative analysis [21,26]. Given the extensive presence of SPECT-CT scanners worldwide [27] and their capability to perform multi-isotope imaging [28], quantitative SPECT-CT studies can be of great importance in patient management, especially for treatment response evaluation [29]. This study aims to demonstrate the important contribution that quantitative SPECT-CT can provide in metastatic bone disease follow-up compared to qualitative assessment regarding changes in radiotracer uptake of the target lesions.

## 2. Materials and Methods

### 2.1. Patients

This is a prospective study in which we collected the data from 172 SPECT-CT studies acquired on 75 female metastatic breast cancer patients with a mean age of 59.31 ± 10.76 who were presenting metastatic bone disease and who underwent at least two bone SPECT-CT studies using [^99m^Tc]-HDP between November 2019 to October 2022 in the Department of Nuclear Medicine of the National Institute of Oncology “Prof. Dr. Alexandru Trestioreanu” Bucharest, Romania. For the present study population, informed consent was obtained after all the patients were instructed about the expected side effects, procedure protocol and radiation exposure. The study was approved by the Ethics Committee of our institution. The main criteria for inclusion were as follows:

- Advanced-stage breast cancer patients, confirmed through biopsy, presenting metastatic bone disease;
- At least one metastatic bone lesion with high radiotracer uptake present in two consecutive scans;
- At least two consecutive SPECT-CT scans within a 6–8-month timeframe;
- Active treatment using osteoclast inhibitors, hormone therapy, chemotherapy, radiotherapy or monoclonal antibody (Table 1);
- Absence of any kind of metal or surgical implants in the field of view (FOV) (because the image artifacts that can influence the quantification accuracy due to erroneous reconstruction and processing);
- Access to measured injected activity, time of measurement, injection time and residual activity in the syringe;
- No statistically significant changes in acquisition parameters between baseline and follow-up study.

### 2.2. Follow-Up Examination

**Figure 1:**
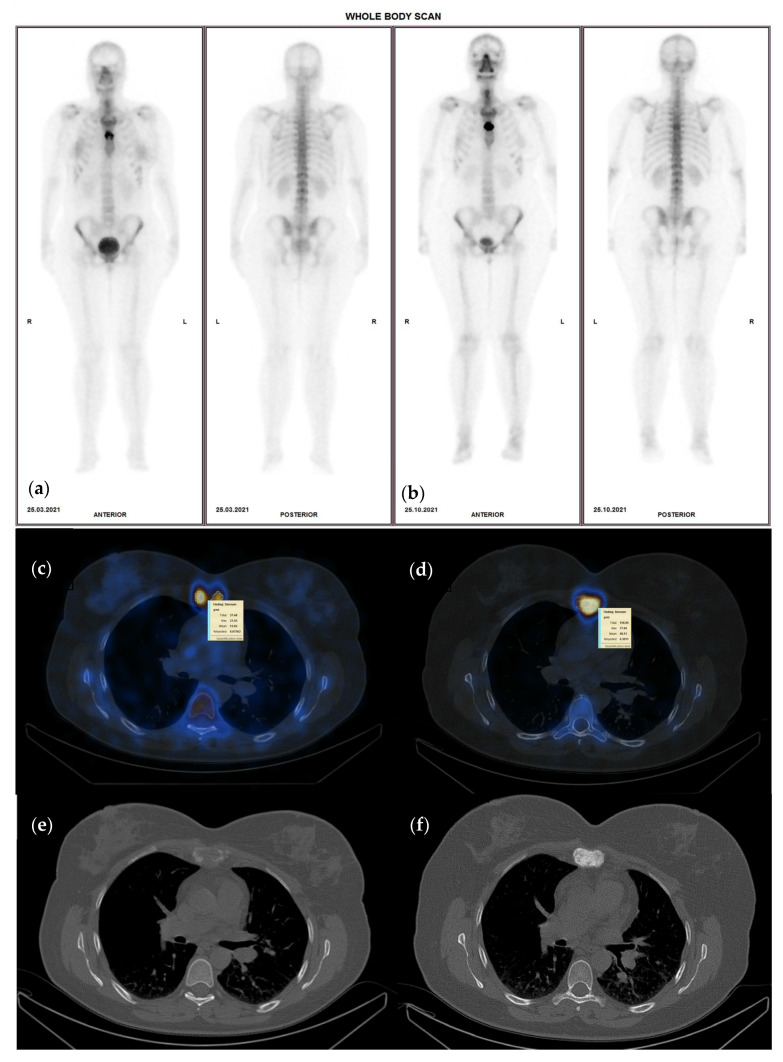
Sixty-nine-year-old female with invasive ductal carcinoma and surgical (right mastectomy), chemotherapy and hormone therapy treatment presenting with bone metastases, represented by high heterogenous radiotracer uptake on the sternum with an SUVmax of 23.5 g/mL (**a**)—WBS and (**c**)—hybrid image corresponding to an osteolytic lesion on CT—(**e**), on the baseline study; (**b**)—WBS, (**d**)—hybrid image of the follow-up SPECT-CT after 8 months of osteoclast inhibitor treatment, presenting higher radiotracer uptake (SUVmax of 77.8 g/mL) although the lesion became osteoblastic on the CT scan (**f**).

A follow-up examination on the metastatic bone patients was performed in our department every 6–8 months after the initial scan to register the changes in radiotracer uptake resulting from the differences in the metabolic activity of the bone metastases. The time interval between treatment and patient scanning was adapted to avoid a possible flare phenomenon that in most cases appeared between 2 to 18 weeks after treatment initiation [30,31]. Concerning radiotherapy, the evaluation of treatment response was conducted no earlier than two months after completion of therapy. These aspects are of great importance in accurately evaluating the treatment response and assessing the extent of osseous involvement. The follow-up examination included WBS and [^99m^Tc] -HDP SPECT-CT with at least two fields of view to ensure all regions of interest were properly evaluated (Figure 1).

### 2.3. SPECT-CT Acquisition Protocol and Reconstruction

Each patient received a [^99m^Tc] -HDP intravenous injection with a mean activity of 659.69 ± 105.03 MBq for the baseline study and 665.65 ± 96.46 MBq for follow-up. The mean injection-to-scan time was 181 min for the first study (S1) and 182 min for the follow-up study (S2). After the intravenous injection of [^99m^Tc] -HDP with a mean value of (9.45 MBq/kg for S1 and 9.60Mbq/kg for S2), SPECT-CT was performed using a GE Discovery DR670 SPECT-CT system with a sensitivity of 160 counts per minute, using low-energy high-resolution collimators. At this point, it is essential to emphasize the reduced variability of the parameters because of their influence on the SUV calculation. In accordance with the European guidelines, we implemented the following SPECT-CT protocol: 128 by 128 matrix size, step and shoot mode, 60 steps, with 20 s per step. For scatter correction, dual-energy window with 140 KeV peak energy ± 10 KeV and 120 ± 5 KeV was chosen. The SPECT scan was completed by a low-dose CT scan performed using 120 kV and 30 mA, dose modulation enabled (GE Smart Scan). CT data were acquired with 3.75 mm slice thickness and then reconstructed in 1.25 mm slices by applying bone enhancement filters provided by the vendor software (BONE PLUS reconstruction filter) to reduce the number of equivocal findings.

SPECT reconstruction was performed using the Ordered Subset Expectation Maximization (OSEM) iterative reconstruction algorithm with 8 subsets and 10 iterations, resolution recovery, scatter correction and attenuation correction based on the attenuation correction map resulting from the CT-acquired data.

### 2.4. Image Interpretation

**Figure 2:**
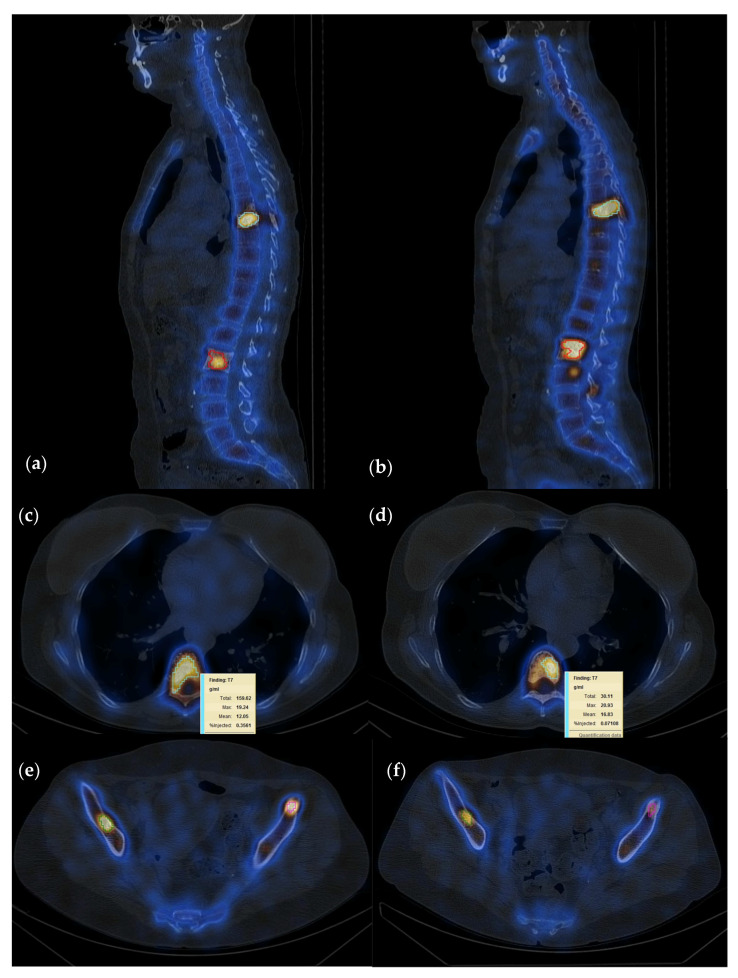
Fifty-five-year-old female patient with invasive lobular carcinoma operated in 2008 (left mastectomy) treated with chemotherapy, radiotherapy and hormone therapy; local recurrence and bone metastasis diagnosed in October 2021; multiple metastatic lesions with high uptake localized at T7, L2, and both iliac spines currently in treatment with osteoclast inhibitors; (**a**–**c**) baseline study; (**d**–**f**) follow-up study after 6 months of osteoclast inhibitor; quantification showed a summed SUVmax 81.3 g/mL on the baseline and 47.2 g/mL on the follow-up study, suggesting PR with a decrease in radiotracer uptake of 58%.

**Figure 3:**
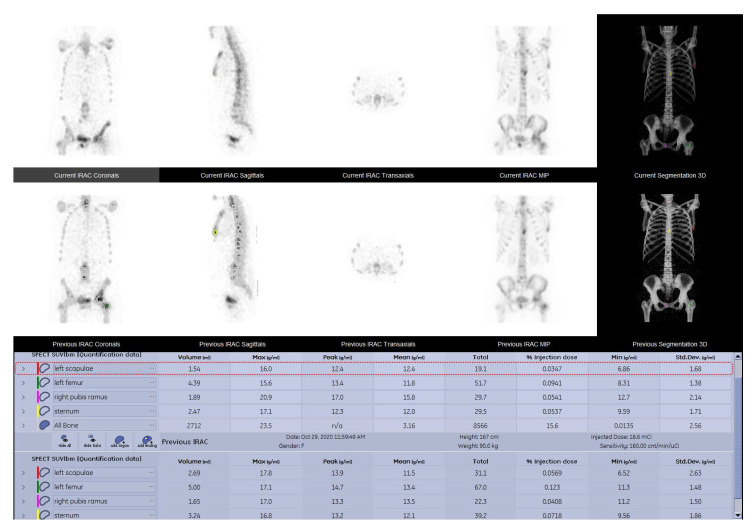
SPECT-CT data quantitative analysis results displayed in comparison between the baseline and the follow up study of a 52-year-old female breast cancer patient with grade two lobular invasive breast carcinoma, presenting multiple metastatic lesions, following chemotherapy and osteoclast inhibitor.

SPECT-CT scans in clinical practice are routinely interpreted using qualitative methods. In our case, two experienced physicians categorized each patient by visual comparison of the uptake in the target lesions on the WBS and SPECT-CT studies in increased, decreased, or equal uptake. In case of disagreement, a common decision was made in a joint meeting. Following the visual assessment of the acquired data and given the fact that there are no clear criteria for SPECT-CT follow-up evaluation, we decided to use the classification method of treatment response evaluation as presented by the currently available positron emission tomography response criteria in solid tumors (PERCIST) 1.0 criteria for quantitative data interpretation, thus categorizing treatment response as follows: Complete Response (CR), Partial Response (PR), Stable Disease (SD) and Progressive disease (PD), with a minimum of 1 and a maximum of 5 lesions being compared following S1 and S2 of the same patient [32]. In patients with less than five bone metastases, all lesions were quantified, while in patients presenting multiple bone metastases, we analyzed five lesions with the highest SUVmax. Contouring of the volumes of interest (VOI) was conducted in a semi-automatic manner using the dedicated segmentation tools provided by the camera vendor (Figure 2). Maximum, peak, mean SUVs (SUVmax, SUVpeak, SUVmean) and tumor volume were calculated for the target lesions (Figure 3). SUVmax was determined as the SUV of the most active voxel within the lesion, SUVpeak as the average activity within an area of one cubic centimeter around the most active voxel and SUVmean as the average SUV of voxels in the volume of interest (VOI). To ensure that the selected lesions were indeed metastatic, we only considered the hypermetabolic foci on SPECT that had a clear correspondent on the CT images. Although the gold standard in lesion characterization and identification is represented by bone biopsy, performing this procedure to every patient is neither practical nor ethical; therefore, morphologic criteria were used as a reference standard in diagnosis for bone metastatic lesions identification [25].

The SUV are used as an absolute unit to express the radiotracer uptake in a VOI. SUV can be normalized to body mass (SUVbw), lean body mass (SUVlbm) or body surface (SUVbs). In our case, SUV normalized by lean body mass was the method of choice. According to data available in the literature, the SUVbw is influenced by the amount of body fat (this being the reason why SUV values are overestimated in obese patients [33,34]), while SUVlbm is not subjected to variations due to body weight or by the amount of lean body mass [33,35,36].

### 2.5. Follow-Up Diagnosis Criteria

In the present study, a baseline scan was performed for all patients before the start of treatment, followed by a second scan at a 6–8-month timeframe. The conclusive diagnosis of disease status was made by comparing the summed SUVmax value of the most active lesions between the baseline and the follow-up study of the same patient (Figure 3) using the following criteria, which were elaborated in agreement to PERCIST as a guideline:

- CR—no activity in any of the lesions on the follow up study;
- PR—more or equal to 30% decrease in summed SUVmax value;
- SD—neither PR nor PD (30% cut-off);
- PD—more than 30% increase of the summed SUVmax value or new tumor foci.

### 2.6. Scanner Calibration

**Figure 4:**
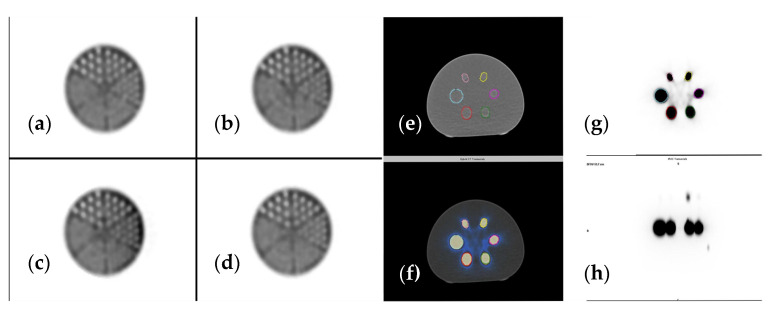
(**a**–**d**) Jaszczak phantom reconstructed using different iterations and subsets, (**e**) CT image (**f**) SPECT-CT image, (**g**) SPECT axial view, (**h**) SPECT sagittal view of NEMA phantom used for accurate scanner calibration.

To ensure accurate measurement in radiotracer uptake of the target lesions, dedicated scanner calibration must be performed. In our case, the SPECT-CT was calibrated using a uniform Jaszczak and a NEMA phantom (Figure 4). After performing scanner calibration, we determined the sensitivity values for both planar and SPECT acquisition.

### 2.7. Statistical Analysis

The data were analyzed using specialized statistical software, SPSS version 26.0 and MedCalc. Both parametric and non-parametric tests were used based on the type of the data included in the variables; therefore, mean comparison of the available data was conducted using Paired Samples T-Test, while correlation relationship between specific data was assessed using either Spearman rho or Pearson r, the first one representing a non-parametric version of the classic Pearson r test used for correlation coefficient determination.

## 3. Results

### 3.1. Lesion Localization

A total of 249 metastatic lesions were assessed, with an average of three lesions per patient (range 1–5 lesions), both from a qualitative and a quantitative point of view and registering the following distribution: 67 (26.90%) of the lesions were identified in the thoracic vertebrae, 55 (22.08%) in the lumbar vertebrae and 60 (24.09%) were localized in the pelvic bones (Table 2). Only 16 (21.33%) of the patients presented solitary bone lesions, while 59 (78.67%) had two or more metastatic bone lesions.

### 3.2. Assessed Parameters Comparison

Following the comparison of the mean values of the assessed parameters for every patient, we obtained statistical results that indicate a significant difference between the values of the SUVmax, SUVpeak, SUVmean and lesion volume measured at the baseline study, compared to the ones registered on the follow-up (Table 3).

### 3.3. Correlation between Qualitative and Quantitative Follow-Up Evaluation

By performing a statistical analysis of the data, specific correlation coefficients were determined between the qualitative assessment of WBS and qualitative SPECT-CT compared to the quantitative analysis of the SPECT-CT data. A correlation coefficient of 0.608 was determined between the qualitative assessment of WBS and quantitative SPECT-CT. This value suggests a moderate grade of correlation between the two variables, indicating that in 60.8% of the cases, the two methods of interpretation positively correlated. Another correlation coefficient was determined between the qualitative and quantitative analysis of the SPECT-CT, with a value of 0.711, indicating a positive strong relationship—the correlation between the two methods being present in 71.1% of the cases (Table 4).

### 3.4. Interpretation of Quantitative SPECT-CT

**Figure 5:**
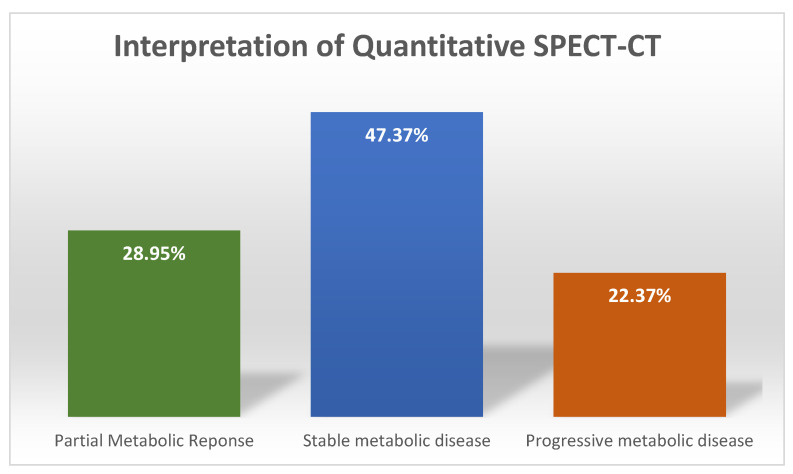
Patient distribution after treatment response evaluation.

The results of the interpretation of the quantitative analysis data using PERCIST criteria for changes in summed SUVmax values as recommendation were as follows: 36 (47.37%) patients registered a change of less than 30%, indicating SD; 22 (28.95%) presented a decrease of more than 30%, indicating PR, while 17 (22.37%) patients had an increase of more than 30%, which corresponds to PD. Only seven patients had new lesions (Figure 5), while none of them had CR.

### 3.5. Correlation of SUVmax with SUVpeak and SUVmean

**Figure 6:**
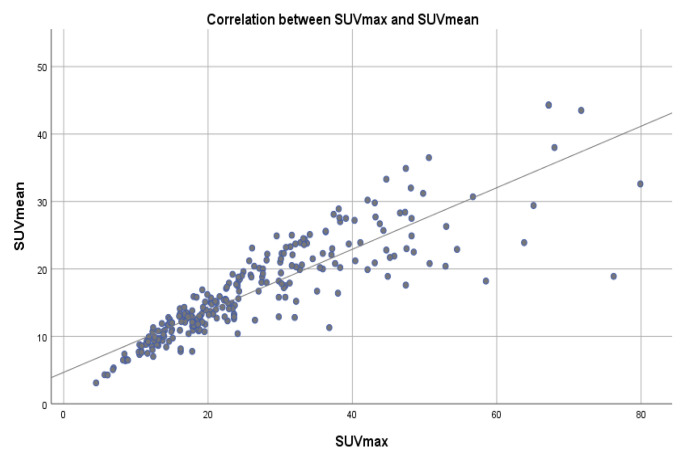
Scatter plot showing the correlation between SUVmax and SUVmean values across a reference line.

**Figure 7:**
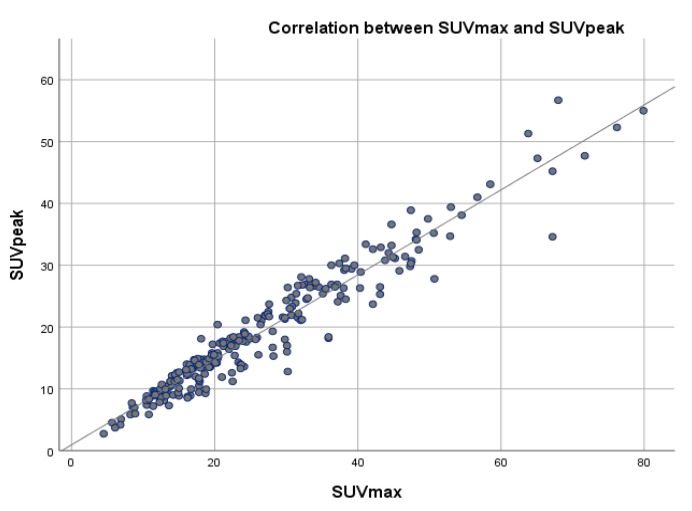
Scatter plot showing the correlation between SUVmax and SUVpeak values across a reference line.

A correlation coefficient between SUVmax and SUVpeak of 0.966 and a correlation coefficient of 0.856 between SUVmax and SUVmean, were determined, suggesting a very strong positive relationship between SUVmax and SUVpeak and strong positive relationship between SUVmax and SUVmean, with a positive correlation being present in 96.6% of the cases regarding SUVpeak and in 87.3% of the cases for SUVmean (Table 5, Figure 6 and Figure 7).

## 4. Discussion

Qualitative analysis has been the main method of imaging interpretation for a long time, offering enough information and accuracy for patient diagnosis and follow-up. Although it represents a solid part of image interpretation, the interobserver variability demonstrated by Beck et.al. [37] and the lack of objective data, mainly for research and patient follow-up, have emphasized the need to develop a quantitative method of molecular imaging analysis that can provide clear, quantifiable and reproducible results. Furthermore, the significant inter- and intra-patient variations in the metabolic uptake of the radiotracer of more than 10 times the average SPECT intensity are not uncommon among patients. Because of this variation, it is difficult to identify a clinically relevant SPECT intensity threshold to identify changes in the target lesions [38]. Furthermore, the integration of the CT scanner with a SPECT camera has led to increased accuracy and has offered the possibility of the morphologic characterization of the target lesions, thus reducing the interobserver variability and number of equivocal lesions. Also, by using the attenuation map resulting from the CT scan, it opens up the possibility for quantitative measurements of radiotracer uptake [39,40].

Quantitative analysis of SPECT-CT data has emerged as an objective method of imaging interpretation following the footsteps of PET-CT, with more and more studies showing the feasibility of performing SUV calculations using SPECT-CT data [20,24,39,40,41,42]. Because the correlation between PET-CT and SPECT-CT SUVs has been demonstrated by Arvola et.al. [43] and because of the fact that both [^99m^Tc] -HDP and [^18^F]-NaF uptake are correlated to the degree of bone remodeling activity of the target lesion, performing an interpretation of SPECT-CT quantitative data using the already existing PET-CT criteria may be a viable option.

By performing quantitative analysis of the SPECT-CT data following the criteria inspired by PERCIST, the results regarding the disease status of every patient on the follow-up scan were determined through an objective, reproducible and more standardized method of imaging interpretation that has the potential to be of great use in performing accurate follow-ups and acquiring data from multicentric studies in the near future—facts previously emphasized by Dickson et al. [24].

The data analysis performed indicated a positive correlation percentage in 60.8% of the cases between WBS and quantitative SPECT-CT and 71.1% between qualitative SPECT-CT and quantitative SPECT-CT, emphasizing the need for implementing a quantitative imaging interpretation method considering that in 48.2% of the cases WBS and in 28.9% of the cases qualitative SPECT-CT failed to accurately determine the state of the disease. For this reason, together with the fact demonstrated by Beck et.al [37] that qualitative interpretation of baseline and follow-up studies yield inconsistent results, performing quantitative interpretation on SPECT-CT data can lead to more accurate patient follow-up.

SUVmax, SUVpeak and SUVmean represent the main data resulting from the quantitative analysis of the SPECT-CT study. Further analysis of the data indicated a very strong positive relationship between SUVmax and SUVpeak, with a correlation coefficient of 0.966 and a strong positive relationship with a correlation coefficient of 0.872 between SUVmax and SUVmean. In a study conducted by Beck et al., similar results were obtained [37], suggesting that in certain cases, such as important SUVmax values variability, both SUVpeak and SUVmean represent a feasible and reliable method of lesion radiotracer uptake evaluation and patient follow-up [44,45]. Based on our results and those reported in the literature, there are strong arguments that emphasize the need to perform a quantitative analysis of SPECT-CT data in metastatic bone patients for longitudinal changes in radiotracer uptake [37,46].

In the last few decades, the development of PET-CT radiotracers has provided the possibility for the evaluation of metastatic bone lesions with higher sensitivity and specificity—the most commonly used being [^18^F]-NaF, with a sensitivity ranging between 95–98% and a specificity of 81–87% registered in studies conducted by Yang et al. and Sheikhbahaei et al. [47,48], which are similar to the ones obtained by Alqahtani et.al regarding bone SPECT-CT diagnostic performances with a sensitivity ranging between 89–94% and specificity of 94–97% [11]. These data indicate that both methods have comparable diagnostic performances in terms of bone metastases evaluation and that SPECT-CT using [^99m^Tc] -HDP remains a suitable option in metastatic bone lesion evaluation given its high sensitivity and specificity, wide availability and good price to quality ratio.

## 5. Limitations

The clinical impact of our findings is hard to be evaluated because of the relatively small patient number and the lack of agreement on radiotracer uptake changes in the quantitative analysis of SPECT-CT data results due to few available data in the literature.

## 6. Conclusion

Quantitative evaluation of SPECT-CT data has the potential to be the first choice of imaging interpretation for patient follow-up and treatment response evaluation, especially for research purposes, because of its objectivity and expression of uptake changes in absolute units. Various data about the target lesions, such as SUVpeak, SUVmean and lesion volume, represent further parameters that can be assessed by performing quantitative analysis of the SPECT-CT data. In addition, reproducibility and need for quantitative data in patient follow-up can be one of the most important arguments in performing SPECT-CT quantitative analysis on a larger scale. Nevertheless, its contribution in clinical practice needs to be demonstrated in further studies among multicentric studies.

## Figures and Tables

**Table 1:** Treatment data of the patients included in the study.

Treatment	No. Patients	Percentage
Chemotherapy	42	55.26%
Hormone therapy	53	69.74%
Radiotherapy	26	34.21%
Osteoclast inhibitors	67	88.16%
Monoclonal antibody	13	17.11%

**Table 2:** Lesion localization.

Region	No. of Lesions	Percentage
Ribs	26	10.44%
Thoracic vertebrae (T)	67	26.90%
Lumbar vertebrae (L)	55	22.08%
Pelvic bones (P)	60	24.09%
Other bone sites(O)	41	16.46%

**Table 3:** Assessed parameters’ mean values comparison.

	Mean Values of Assessed Parameters			
	Tumor volume	SUVmax	SUVpeak	SUVmean
Baseline	5.14 ± 9.28	25.45 ± 14.04	19.13 ± 10.11	16.74 ± 7.34
Follow-up	5.91 ± 10.65	21.79 ± 12.07	16.39 ± 9.12	14.17 ± 6.16
Statistical sig.	*p* < 0.05	*p* < 0.05	*p* < 0.05	*p* < 0.05

**Table 4:** Correlation coefficient and statistical significance between qualitative and quantitative imaging interpretation methods.

	Quantitative SPECT-CT vs. Qualitative WBS	Quantitative SPECT-CT vs. Qualitative SPECT-CT
Correlation Coefficient (Spearman rho)	0.608 **	0.711 **
Statistical significance (*p* value)	*p* < 0.001	*p* < 0.001

**. Correlation is significant at the 0.01 level (2-tailed).

**Table 5:** SUVmax and SUVmean correlation coefficient and statistical significance.

		SUVmean	SUVpeak
SUVmax	Pearson Correlation	0.856	0.966
	Significance	*p* < 0.001	*p* < 0.001
	N	249	249

Correlation is significant at the 0.01 level.

## Data Availability

All data generated or analyzed during this study are included in the manuscript.
